# Importance of early follow‐up of *Mycoplasma pneumoniae* pneumonia in pediatric patients: A case series

**DOI:** 10.1002/pdi3.27

**Published:** 2023-09-07

**Authors:** Hua Deng, Qin Xie, Wenjie Wu

**Affiliations:** ^1^ Department of Pediatrics Chongqing Youyoubaobei Women and Children's Hospital Chongqing China

**Keywords:** bronchiolitis obliterans, early diagnosis, *Mycoplasma pneumoniae*, pediatrics

## 1 INTRODUCTION

*Mycoplasma pneumoniae* is the leading cause of pediatric community‐acquired pneumonia. Data indicate that *Mycoplasma pneumoniae* pneumonia (MPP) with bronchiolitis is closely related to asthma and bronchiolitis obliterans (BO).^1^ Nonoptimal timing of treatment increases the risk of progression to BO. Thus, early follow‐up and intervention following the acute phase of MPP are necessary to prevent complications. Herein, we describe the treatment and outcomes of three such cases, suggesting early intervention and further research to provide clinical foundations for appropriate treatment strategies.

## 2 CASE PRESENTATIONS

We retrospectively analyzed the data of three patients admitted between January 2022 and September 2022 with a diagnosis of MPP with bronchiolitis. Their medical histories and laboratory examination results are presented in Table 1. Medical histories were otherwise nonspecific. Blood and sputum culture results were negative, and biochemical parameters were within normal limits.

**TABLE 1:** Medical history and laboratory results of the three patients.

Patients		Case 1	Case 2	Case 3
Age (years)		7	7	7
Sex		Male	Male	Male
Symptoms	Fever	High fever for 4 days	A slight fever	High fever for 3 days
	Cough	Strong, dry, and paroxysmal cough	Severe cough and yellow‐green phlegm for 10 days	Persistent and paroxysmal cough for 2 weeks
	Others	Chills	Tolerable pain in the right chest	None
Past history		Rhinitis	Eczema and capillary bronchitis	Atopic dermatitis
Prior allergic reactions		None	Milk, peanuts, and eggs	None
Family histories		None	Rhinitis	None
Physical examination	Tachypnea	Yes	Any significant findings	None
	Pulmonary rales	Few moist rales in the right lung		Few moist rales in the right lung
Laboratory tests	White blood cells (WBC) (*10^6^ cells/L)	5820	23,360	6670
	Neutrophil (%)	57	86	65
	Eosinophil (%)	10	5	4
	C‐reactive protein (CRP) (mg/dL)	18.2	55	12.1
	Procalcitonin (PCT) (ng/ml)	1.49	Normal	0.11
	Lactate dehydrogenase (LDH) (U/L)	270.2	Normal	359.6
	*MP* polymerase chain reaction (PCR)	Positive	None	Positive
	MP‐IgM	None	1:320	None
Lung function at discharge	FEV1 (%pred)	85.4	69.9	85.9
	FEF50 (%pred)	63.2	51.6	64.7
	FEF75 (%pred)	64.6	46.9	48.5
	MMEF (%pred)	65.8	48.3	56.5
	Dilation test	Negative	Positive	Positive
Convalescent lung function		Normal	Abnormal	Normal

### 2.1 Case 1

A 7‐year‐old boy had fever and cough that persisted for 4 days. The middle lobe of the right lung was consolidated based on high‐resolution computed tomography (HRCT) images. Bronchial wall thickening, tree bud signs, and lobular nodules were present in both lungs. The boy was admitted and administered intravenous methylprednisolone (1 mg/kg/day) for 3 days and azithromycin for 7 days.

At the time of discharge, the patient had occasional productive cough and exertional fatigue. He demonstrated more prominent wet rales in the right lung and a protracted expiratory phase. Pulmonary function tests revealed small‐airway dysfunction, a bronchodilator challenge was nonproductive, and HRCT showed mostly resolved lung consolidation but widespread bronchiolitis. Although the in situ infection was effectively controlled, the lesion characteristics and distribution expanded to those of diffuse bronchiolitis in both lungs. This suggested that the infection led to widespread secondary lung inflammation, causing exertional fatigue and delayed regression of lung signs.

The patient was advised to complete courses of azithromycin and oral prednisone for 10 days. Thereafter, the patient had no cough but still experienced exhaustion, and physical examination revealed absence of lung rales. At 28 days and again at 4 months after discharge, the patient reported no discomfort, and the HRCT and pulmonary function test results had returned to normal.

### 2.2 Case 2

A 7‐year‐old boy was admitted with a 10‐day history of cough. HRCT revealed substantial consolidation in the middle lobe of his right lung and extensive uneven exudation. He received courses of oral azithromycin for 3 days and intravenous amoxicillin‐clavulanate for 6 days, along with budesonide atomization for 1 week. Lung function and HRCT were not examined at discharge. Within 2 months of discharge, cough and wheezing recurred, and a few medium‐fine moist rales could be heard on multiple auscultations. Pulmonary function testing revealed small‐airway dysfunction with no improvement after bronchodilator therapy. HRCT demonstrated a mosaic perfusion pattern, and the lesion distribution was consistent with that of the acute phase. The patient was considered to have postinfectious bronchiolitis obliterans (PIBO), and although lung function returned to normal, HRCT findings did not significantly improve after retreatment for 3 months.

### 2.3 Case 3

A 7‐year‐old boy was admitted with a 2‐week history of cough. HRCT revealed features of diffuse bronchiolitis in the anterior basal segments of the lower lobes with solid nodular shadows.

The patient received combined therapy with azithromycin and ceftriaxone for 5 days, and the cough was significantly relieved at discharge. However, 1 week later, he experienced severe coughing after exercise with relief following rest, and moderate moist rales were audible on auscultation. Pulmonary function testing revealed small‐airway dysfunction that improved after bronchodilator therapy. The patient was considered to have asthma and was prescribed Seretide 100 μg inhalation twice daily. HRCT conducted after 1 month revealed disappearance of the inflammation. Meanwhile, pulmonary function returned to normal.

## 3 DISCUSSION

*M. pneumoniae* infection correlates epidemiologically with asthma and PIBO.^1^ These relationships have been the focus of research; however, many clinical problems remain. For example, when BO is diagnosed in nonsevere MPP cases, the optimal time for treatment has long passed. Thus, it is crucial to monitor for asthma and BO in the early stages after MPP and to appropriately intervene to prevent complications.

The pathophysiology of MPP involves the immunological inflammatory response and cellular damage due to direct invasion. The effectiveness of antibiotics in treating MPP is limited, and the prognosis of MPP may be influenced by the host's immunological response.^2^ *M. pneumoniae‐g*enerated community‐acquired respiratory distress syndrome toxin can cause a 30‐fold increase in the production of the Th‐2 chemokines, IL‐4, and IL‐13, which are associated with the Th‐2 immune response. The inflammatory response is amplified if the host has had allergies or wheezing. This inflammation may have contributed to the recurrent or persistent cough, nonresolution of signs, and prolonged course of disease in our three cases due to bronchial hyperresponsiveness or persistent airway inflammation. However, the duration of inflammatory injury remains unclear.^1^

Acute bronchiolitis is associated with the incidence of PIBO, and the lesional distribution matches that of bronchiolitis.^3^ Partial or total obstruction of the airway lumen results from the inflammatory development of granulation or fibrosis over time. According to Huang et al.,^4^ the most reliable indicator of PIBO risk is pulmonary rales lingering for ≥7.5 days. In Case 1, pulmonary moist rales did not decrease for more than 1 week after effective treatment. However, because we monitored the bronchiolitis using HRCT and the small airway impairment using pulmonary function testing, and provided active treatment, the prognosis was favorable following management. Additionally, a lack of regular follow‐up and intervention led to BO in Case 2.

Both PIBO and asthma involve pathology of the small airways. MPP‐induced small airway damage should be the focus when determining the prognosis of these illnesses. In addition to HRCT, pulmonary ventilatory function markers, including FEF_50_, FEF_75_, and MMEF, can identify small airway injuries. The common imaging features of bronchiolitis include bronchial wall hypertrophy, tree‐in‐bud signs, patchy centrilobular nodules, and unevenly distributed inflammation. Studies have demonstrated that children with MPP in the acute stage have varying degrees of major and small airway functional impairments. Most children, as in our three cases, experience considerably improved lung function throughout the recovery period; however, recovery is slower for the small airways, which are more severely damaged.^5^ Jung et al.^1^ discovered that infectious bronchiolitis and impaired pulmonary function at the time of initial diagnosis were risk factors for a poor prognosis. To improve the prognosis of MPP, we recommend follow‐up pulmonary imaging and pulmonary function testing of high‐risk groups before and after discharge.

The strength of our study is that, unlike other studies that focused on the treatment of BO, we have emphasized early monitoring and prevention. Our study also has certain limitations. Despite the diagnosis of MPP in these three patients, unrelated medications were also administered because of the possibility of mixed infections. In addition, we reported only three cases; determining the optimal management of MPP after the acute stage requires statistical analysis of large case samples.

Follow‐up after the acute period of MPP is crucial. Complications of MPP could potentially be lessened by comprehensive assessment of small airway damage and early detection of persistent airway inflammation and structural changes by documenting a personal history of allergy or wheezing, HRCT findings, and pulmonary function test results prior to discharge and regression of pulmonary signs.

## AUTHOR CONTRIBUTIONS

DH was involved in conceptualization, data curation, writing, and investigation. QX helped in data curation, review, and editing. WJ contributed to conceptualization, supervision, and validation.

## CONFLICT OF INTEREST STATEMENT

The authors declare no conflicts of interest.

## ETHICS STATEMENT

The present study complied with the ethical principles of the Helsinki Declaration of the World Medical Association and has been approved by the ethics committee of the Chongqing Youyoubaobei Women and Children's Hospital.

## CONSENT FOR PUBLICATION

The patients' legal guardians provided informed consent for publishing the detailed information (including pictures) of the patients.

## Data Availability

All data included in this study are available upon reasonable request to the corresponding author.

## References

[1] Jung JH , Kim GE , Min IK , et al. Prediction of postinfectious bronchiolitis obliterans prognosis in children. Pediatr Pulmonol. 2021;56(5):1069‐1076. 33305910 10.1002/ppul.25220

[2] Yang EA , Kang HM , Rhim JW , Kang JH , Lee KY . Early corticosteroid therapy for *Mycoplasma pneumoniae* pneumonia irrespective of used antibiotics in children. J Clin Med. 2019;8(5):726. 31121867 10.3390/jcm8050726 PMC6572103

[3] Zhao C , Liu J , Yang H , Xiang L , Zhao S . *Mycoplasma pneumoniae*‐associated bronchiolitis obliterans following acute bronchiolitis. Sci Rep. 2017;7(1):8478. 28814783 10.1038/s41598-017-08861-7 PMC5559585

[4] Huang K , Liu J , Lv W , et al. Analysis of risk factors of bronchiolitis obliterans in children with *Mycoplasma pneumoniae* bronchiolitis. Comput Math Methods Med. 2022;2022:9371406. 35242215 10.1155/2022/9371406 PMC8886696

[5] Leng J , Yang Z , Wang W . Diagnosis and prognostic analysis of *Mycoplasma pneumoniae* pneumonia in children based on high‐resolution computed tomography. Contrast Media Mol Imaging. 2022;2022:1985531‐1985537. 35542756 10.1155/2022/1985531 PMC9054457

